# Regulator of G-protein signaling 5 regulates the shift from perivascular to parenchymal pericytes in the chronic phase after stroke

**DOI:** 10.1096/fj.201900153R

**Published:** 2019-04-30

**Authors:** Michaela Roth, Abderahim Gaceb, Andreas Enström, Thomas Padel, Guillem Genové, Ilknur Özen, Gesine Paul

**Affiliations:** * Translational Neurology Group, Department of Clinical Science, Lund University, Lund, Sweden;; † Department of Medicine, Integrated Cardio Metabolic Centre, Karolinska Institute, Huddinge, Sweden;; ‡ Department of Neurology, Scania University Hospital, Lund, Sweden; § Wallenberg Centre for Molecular Medicine, Lund University, Lund, Sweden

**Keywords:** PDGFR-β, ischemia, vascular remodeling

## Abstract

Poststroke recovery requires multiple repair mechanisms, including vascular remodeling and blood-brain barrier (BBB) restoration. Brain pericytes are essential for BBB repair and angiogenesis after stroke, but they also give rise to scar-forming platelet-derived growth factor receptor β (PDGFR-β)–expressing cells. However, many of the molecular mechanisms underlying this pericyte response after stroke still remain unknown. Regulator of G-protein signaling 5 (RGS5) has been associated with pericyte detachment from the vascular wall, but whether it regulates pericyte function and vascular stabilization in the chronic phase of stroke is not known. Using RGS5–knockout (KO) mice, we study how loss of RGS5 affects the pericyte response and vascular remodeling in a stroke model at 7 d after ischemia. Loss of RGS5 leads to a shift toward an increase in the number of perivascular pericytes and reduction in the density of parenchymal PDGFR-β–expressing cells associated with normalized PDGFR-β activation after stroke. The redistribution of pericytes resulted in higher pericyte coverage, increased vascular density, preservation of vessel lengths, and a significant reduction in vascular leakage in RGS5-KO mice compared with controls. Our study demonstrates RGS5 in pericytes as an important target to enhance vascular remodeling.—Roth, M., Gaceb, A., Enström, A., Padel, T., Genové, G., Özen, I., Paul, G. Regulator of G-protein signaling 5 regulates the shift from perivascular to parenchymal pericytes in the chronic phase after stroke.

Stroke is one of the leading causes of mortality worldwide. Ischemic stroke results in a complex spatial and temporal cascade of events. In the acute phase, the stop in blood flow leads to lack of oxygen and glucose followed by rapid cell death and the breakdown of the blood-brain barrier (BBB) (1). There is an increasing interest in the role of pericytes in ischemic stroke, especially their role in BBB leakage and vascular damage. Most studies, however, have been limited to the acute phase of stroke (2, 3). The few reports examining the role of pericytes in the chronic phase suggest that a subpopulation of vessel wall–derived pericytes migrates into the injured parenchyma to become scar-forming pericytes (4, 5). However, poststroke recovery during the chronic phase requires remodeling of the vasculature (6), a process that is essential for vascular repair and BBB restoration (7). The remodeling of the vasculature is closely associated with vessel stabilization by brain pericytes (8–11). How this process is regulated is much less studied, and only a few molecular players behind poststroke vascular repair through pericytes have been identified (10–13). Especially, how the fate choice from vascular to scar-forming pericytes is regulated is not known. Upon ischemia, pericytes change from a quiescent state to an activated state (14), a process that requires alterations in the expression pattern of different proteins. Regulator of G-protein signaling 5 (RGS5), a negative regulator of GPCR signaling, is exclusively expressed in the brain by pericytes (15, 16). Recently, RGS5 has been identified as an ischemia-inducible factor that is up-regulated in activated pericytes (14, 17, 18). Pericytes that acutely detach from the vascular wall after stroke have been shown to express RGS5 (14), indicating that pericyte detachment may be regulated by RGS5. Recruitment of pericytes to endothelial cells is mediated by, for example, platelet-derived growth factor BB (PDGF-BB) secreted by endothelial cells, which binds to PDGF receptor β (PDGFR-β) that is expressed on pericytes (19). PDGF-BB and its receptor PDGFR-β are both up-regulated upon stroke (4, 20–22). RGS5 has been suggested to regulate PDGFR-β signaling *in vitro* (23); however, its potential role in the regulation of PDGFR-β signaling in pericytes and its impact on vascular remodeling in the chronic phase after stroke remains elusive.

Here we investigate the effect of RGS5 on pericytes in the chronic phase after stroke and demonstrate that lack of RGS5 in pericytes clearly modifies the pericyte response at 7 d after stroke. We show that loss of RGS5 changes the spatial distribution of PDGFR-β^+^ cells in the ischemic core toward an increase in the number of perivascular pericytes and a decrease in the density of parenchymal PDGFR-β^+^ cells, whereas it retains PDGFR-β signaling at baseline levels comparable with sham-treated controls. We demonstrate that RGS5–knockout (KO) mice have higher pericyte coverage of blood vessels, preservation of vessel length, and a significant reduction in vascular leakage at 7 d after stroke compared with wild-type (WT) mice.

Our data demonstrate that RGS5 in brain pericytes plays an important role in modulating the pericyte response to stroke. RGS5 may be a future target to enhance vascular repair and reduce BBB leakage after ischemia.

## MATERIALS AND METHODS

### Animals

In this study, we used a KO/knock-in reporter mouse strain that expressed green fluorescent protein (GFP) under the promoter of RGS5 in a C57bl/6 background (24). We used 10-wk-old male *Rgs5^gfp/gfp^* mice (referred to as RGS5-KO mice) and WT mice (*Rgs5^+/+^*, referred to as WT) as control mice. To visualize activated pericytes, we used *Rgs5^gfp/+^* heterozygous mice (referred to as RGS5-HET) as a control. In RGS5-HET mice, one of the alleles of RGS5 is replaced by GFP, making it possible to track pericytes by GFP expression under the activated RGS5 promoter. In RGS5-KO mice, both alleles of RGS5 are replaced by GFP, whereby only GFP is expressed upon RGS5 promoter activity, but no RGS5 protein is produced. RGS5-KO mice have previously been extensively validated and characterized and shown to be viable and fertile and to develop without obvious defects (24). GFP was demonstrated to be selectively expressed in pericytes (24), and no changes in pericyte numbers or vascular density were observed under physiologic conditions in the cortex of these mice (17).

Animals were housed under standard conditions, with access to food and water *ad libitum*. All experimental protocols were approved by the ethical committee of Lund University, and all methods were carried out in accordance with the relevant guidelines and regulations.

### Permanent middle cerebral artery occlusion

The distal part of the left middle cerebral artery (MCA) was permanently occluded to induce a focal cerebral ischemia, as previously described in Llovera *et al*. (25). In brief, animals were anesthetized with isoflurane and an incision was made between the left ear and eye. The temporal muscle was detached from the skull in its apical and dorsal part. A small craniotomy was made with a surgical drill above the anterior distal branch of the MCA, located in the rostral part of the temporal area, dorsal to the retro-orbital sinus. After exposure of the MCA, it was permanently occluded by electrocoagulation using an electrosurgical unit (ICC 50; Erbe, Tübingen, Germany). Marcaine (AstraZeneca, Gothenburg, Sweden) was locally applied and the wound was sutured. Sham-treated animals were treated the same way but without ligation of the MCA.

### Tissue processing

Mice were euthanized at 7 d after permanent MCA occlusion (pMCAO) and transcardially perfused with PBS followed by 4% paraformaldehyde. The brains were removed and placed in 4% paraformaldehyde and postfixed overnight. They were then placed in 30% sucrose in PBS for 1 d and sectioned in coronal sections of 40 μm in 12 series.

### Vessel stability

To assess vessel leakage, 0.1 ml of 2% Evans blue (MilliporeSigma, Burlington, MA, USA) was injected 2 h prior to saline perfusion into the tail vein. Mice were transcardially perfused with saline, and brains were stripped on ice and weighed. Each sample was homogenized in 25% trichloroacetic acid solution, kept at 4°C overnight, and then centrifuged for 30 min at 1000 *g* at 4°C. The Evans blue content in 100 μl of supernatant was then measured at 620 nm using a 96-well plate reader. All values were within the standard curve, consisting of diluted Evans blue in 1× PBS in the range from 1 to 100 ng/ml (*R* = 0.98).

### Immunohistochemistry

Brain sections were washed 3 times in PBS for 5 min and then blocked for 30 min in 5% normal donkey or goat serum in 0.25% Triton X-100 (Alfa Aesar, Haverhill, MA, USA) in PBS. Primary antibodies were incubated overnight at room temperature in 3% serum in 0.25% Triton X-100 in PBS. For PDGFR-β detection, sections were pretreated with citrate buffer for 20 min at 80°C. The following primary antibodies were used: chicken anti-GFP (1:5000; Abcam, Cambridge, MA, USA), goat anti-podocalyxin (1:400; R&D Systems, Minneapolis, MN, USA), rabbit anti–PDGFR-β (1:200; Cell Signaling Technology, Danvers, MA, USA), rat anti-CD13 (1:100; Bio-Rad, Hercules, CA, USA), rabbit anti-fibrinogen (1:400; Abcam), and mouse anti Neuronal nuclei (NeuN) (1:500; MilliporeSigma).

For immunofluorescence, sections were washed with PBS, and the staining was visualized using species-specific fluorophore-conjugated or biotin-conjugated (Thermo Fisher Scientific, Waltham, MA, USA) secondary antibodies.

For bright-field stainings, sections were quenched with a peroxidase solution (3% H_2_O_2_, 10% methanol, diluted in PBS) for 15 min prior to blocking. After incubation with the primary antibody, sections were incubated for 2 h with corresponding biotinylated secondary antibodies (1:200; Vector Laboratories, Burlingame, CA, USA), followed by 1 h of signal enhancement using an avidin-biotin kit (Vectastain Elite ABC Kit; Vector Laboratories); they were revealed using chromogen 3,3-diaminobenzidine (DAB Peroxidase Substrate Kit; Vector Laboratories).

### Image processing and cell counting

Fluorescent immunostainings were visualized using a Leica SP8 confocal microscope (Leica Microsystems, Wetzlar, Germany).

To quantify cell numbers, cells were counted on ×63 and ×20 magnification confocal pictures spanning the defined area in the infarct core area. The infarct core was defined according to initial assessment of PDGFR-β staining, which showed that the infarct core had a dense network of PDGFR-β staining. Pictures in the contralateral hemisphere were taken in the same area as the infarct area on the ipsilateral hemisphere. The areas were further confirmed by NeuN staining, which showed that the infarct core had almost complete loss of NeuN^+^ cells. Either three ×63 images or one ×20 image per region of interest was taken, for 3 subsequent sections and averaged per animal. Pictures were analyzed with ImageJ (National Institutes of Health, Bethesda, MD, USA). Pericyte numbers were counted as cells that were positive for a pericyte marker with a DAPI^+^ nucleus and a perivascular location around the capillaries (under 10 μm in diameter). The analysis of the density of PDGFR-β and the vasculature was performed as previously described (17). Briefly, the area covered by either PDGFR-β or podocalyxin was analyzed using the ImageJ area measurement tool, with which pictures were subjected to threshold processing and produced a binary image. The density was expressed as a percentage of the total area analyzed. For pericyte coverage, GFP^+^ pericytes and podocalyxin^+^ blood vessels were separately subjected to threshold processing, and pericyte coverage was determined as the percentage of the GFP^+^ pericyte area covering the podocalyxin^+^ capillary surface.

Figures were assembled in Adobe Illustrator CS5 v. 15.0.0 (Adobe, San Jose, CA, USA).

### Western blot analysis

For Western blot analysis, mice were perfused transcardially with PBS, and the brain was removed. The infarct core and corresponding area in sham-treated animals were dissected and kept at −80°C. The tissue was cut into small pieces and suspended in RIPA buffer (Thermo Fisher Scientific) with protease inhibitor (Thermo Fisher Scientific) and homogenized with Lysing Matrix D (MP Biomedicals, Santa Ana, CA, USA). Protein concentrations were evaluated with the Pierce BCA Protein Assay Kit (Thermo Fisher Scientific). Fifty µg of each sample was incubated in Laemmli buffer (Bio-Rad, Hercules, CA, USA) (5 min at 100°C) and resolved by SDS-PAGE (Bio-Rad). Membranes were analyzed using rabbit anti–PDGFR-β (1:1000) and rabbit anti-phosphorylated PDGFR-β Tyr751 (1:1000; Cell Signaling Technology). Images were acquired using the ChemiDoc MP Imaging System (Bio-Rad) and analyzed with ImageJ. Because of the number of samples, several gels were run simultaneously and processed in parallel. The same membranes were used to compare total PDGFR-β protein with pPDGFR-β.

### Stroke size measurement

Whole series of NeuN staining were scanned with a high-resolution scanner. In ImageJ, the contralateral hemisphere, ipsilateral hemisphere, and infarct area were outlined, and their area was measured. The infarct volume was calculated subsequently. The percentage of infarct volume was calculated as 100 × [(*V*_contralateral hemisphere_ − *V*_ipsilateral hemisphere without infarct_)/*V*_contralateral hemisphere_], and the percentage of swelling was calculated as 100 × [(*V*_ipsilateral hemisphere_ − *V*_contralateral hemisphere_)/*V*_ipsilateral hemisphere_].

### Statistics

GraphPad Prism v. 7.0c (GraphPad Software, La Jolla, CA, USA) was used for statistical analysis of the data. Data are expressed as means ± sd, and the given *n* values represent the number of animals used. For 2-group comparison, Student’s *t* test was used, and for multiple-group comparison, 2-way ANOVA followed by Tukey’s multiple comparisons test was used. Significance was set at *P* < 0.05.

## RESULTS

### PDGFR-β^+^ cells are mainly associated with the vasculature in RGS5-KO mice after stroke

**Figure 1:**
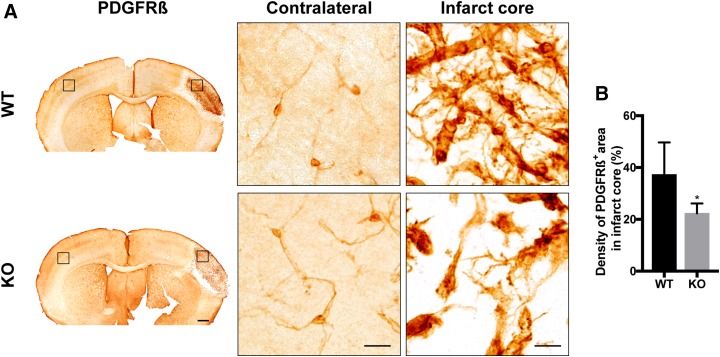
Loss of RGS5 results in reduced density of PDGFR-β^+^ cells. *A*) Representative bright-field images of 3,3-diaminobenzidine staining of PDGFR-β at 7 d after pMCAO of WT and RGS5-KO mice. Left panel shows overview of sections. Black boxes show location of higher-magnification pictures. Middle panel shows higher magnification of contralateral hemisphere and right panel shows higher magnification of the infarct core. *B*) Quantification of the density of PDGFR-β^+^ area in the infarct core at 7 d after stroke. *n* = 6; data are represented as means ± sd. Scale bars, 500 µm (overview), 10 μm (higher magnification). *P < 0.05, Student’s *t* test.

We and others have previously shown that parenchymal PDGFR-β^+^ cells in the infarct area progressively increase with time after the ischemic injury (4, 14, 20). First, we asked whether loss of RGS5 leads to changes in the morphology of PDGFR-β^+^ cells at 7 d after stroke. In the contralateral hemisphere, PDGFR-β expression was only found in cells with typical pericyte morphology (**Fig. 1*A***). In the infarct area, however, PDGFR-β^+^ cells showed differences in morphology and tissue distribution between genotypes (Fig. 1*A*). In both WT and RGS5-KO mice, the infarct area was densely packed with PDGFR-β–expressing cells with irregular cell projections. Quantitative analysis showed that the density of the PDGFR-β^+^ area was significantly lower in RGS-KO mice compared with WT mice (KO 22.4 ± 3.8%; WT 37.3 ± 12.4%; *P* = 0.036) (Fig. 1*B*).

**Figure 2:**
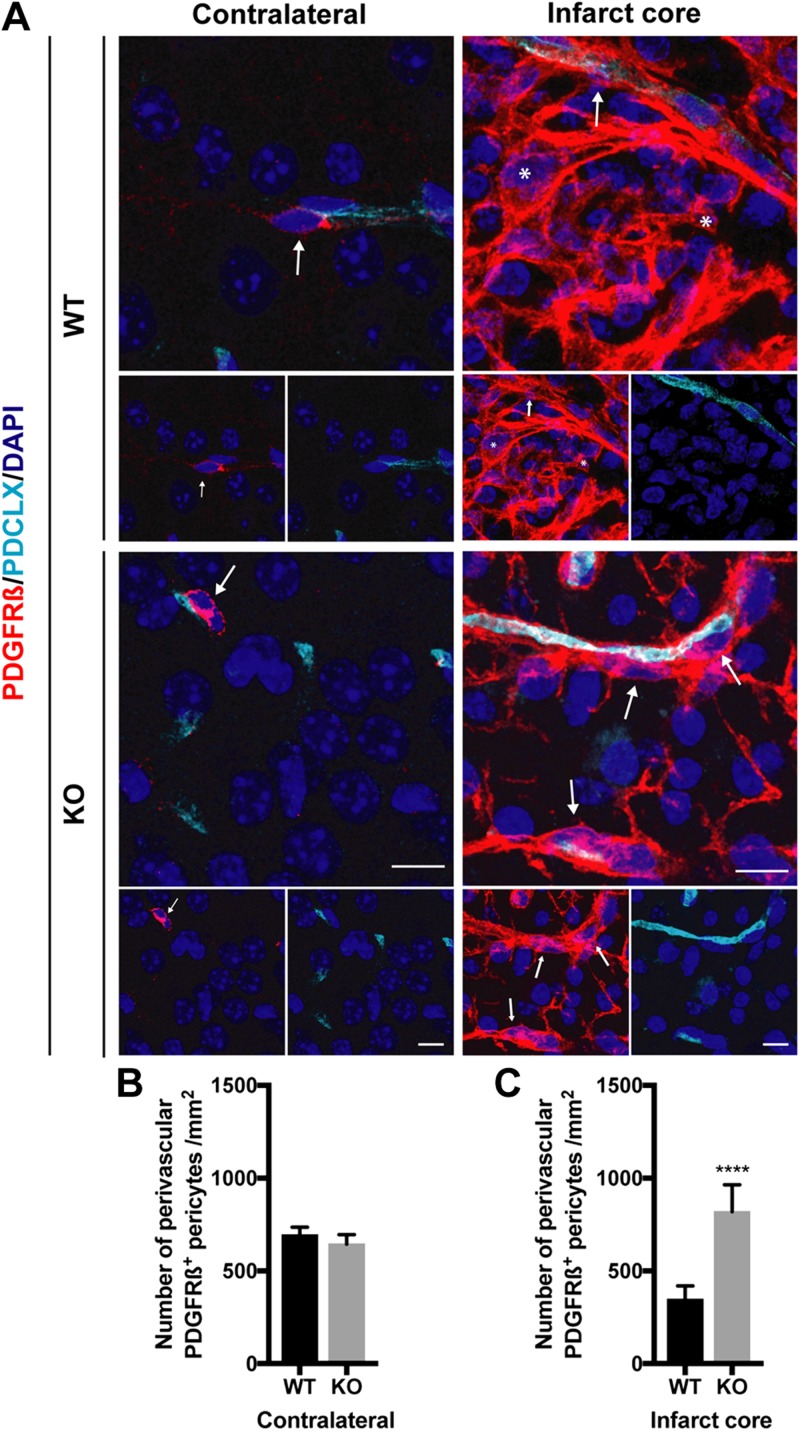
Increased numbers of perivascular PDGFR-β^+^ cells in RGS5-KO mice. *A*) Representative confocal images of PDGFR-β (red) and the vasculature marker podocalyxin (PDCLX, cyan) of the contralateral hemisphere (left panel) and the infarct core (right panel) of WT and KO mice 7 d after stroke. Arrows indicate perivascular PDGFR-β^+^ cells, and asterisks indicate parenchymal PDGFR-β^+^ cells. *B*) Quantification of the number of perivascular PDGFR-β^+^ pericytes in the contralateral hemisphere. *C*) Quantification of the number of perivascular PDGFR-β^+^ pericytes in the infarct core. *n* = 6; data are represented as means ± sd. Scale bars, 10 μm. ****P < 0.0001, Student’s *t* test.

We next investigated the spatial relation of PDGFR-β^+^ cells to the capillaries in the infarct area using confocal imaging (**Fig. 2**). In the contralateral hemisphere of both genotypes, all PDGFR-β^+^ cells had a pericyte morphology, were located around blood vessels, and their numbers did not differ between genotypes (Fig. 2*A, B*). Within the infarct core, however, there were 2 groups of PDGFR-β–expressing cells: the first group was found in the parenchyma (distant from the capillaries) and showed an amoeboid-like morphology with irregular cell projections, here classified as parenchymal PDGFR-β^+^ cells; the second group had a typical pericyte morphology and was found around capillaries, here classified as perivascular PDGFR-β^+^ cells (Fig. 2*A*). We observed both parenchymal and perivascular PDGFR-β^+^ cells in the infarct core of both genotypes, but the density of parenchymal PDGFR-β^+^ cells was lower in the infarct core of RGS-KO mice as compared with WT mice (see also Fig. 1), whereas the number of perivascular PDGFR-β^+^ cells was significantly higher in RGS5-KO mice compared with WT mice (KO 820 ± 145 cells/mm^2^; WT 347 ± 72 cells/mm^2^; *P* = 0.0001) (Fig. 2*C*), indicating a shift in the spatial distribution of PDGFR-β^+^ cells.

### Phenotypes of perivascular and parenchymal PDGFR-β^+^ cells

**Figure 3:**
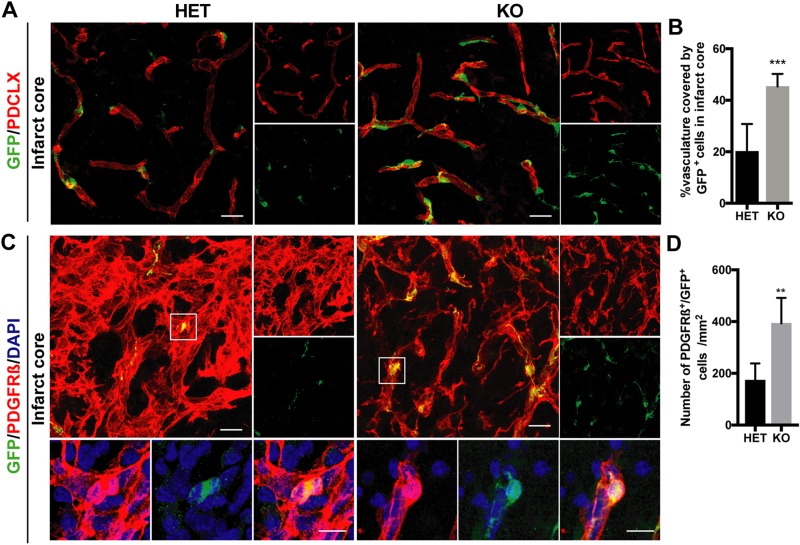
RGS5-KO mice have higher pericyte coverage 7 d after stroke. *A*) Representative confocal pictures of the infarct core of GFP^+^ pericytes (green) and the vasculature [podocalyxin (PDCLX), red]. Right panels show single channels of PDCLX and GFP, respectively. *B*) Quantification GFP^+^ pericyte coverage of the vasculature in the infarct core. *C*) Representative confocal pictures of the infarct core of PDGFR-β (red) and GFP (green) staining. Boxes indicate where higher-magnification pictures were taken. Right panels show single channels of PDGFR-β and GFP staining, respectively. Lower panels show higher magnification of pericytes colabeling for PDGFR-β and GFP. *D*) Quantification of the numbers of PDGFR-β^+^ and GFP^+^ pericytes in RGS5-HET and RGS5-KO mice in the infarct core. *n* = 6; data are represented as means ± sd. Scale bars, 20 μm (10 μm in higher magnification). **P < 0.01, ***P < 0.001, Student’s *t* test.

Having observed that PDGFR-β^+^ cells were mainly associated with the vasculature in the infarct core of RGS5-KO mice, we next characterized the PDGFR-β^+^ cells around the blood vessels. To do that, we investigated the relation between perivascular PDGFR-β^+^ cells and GFP^+^ cells and analyzed the number of PDGFR-β^+^ cells colabeling with GFP. GFP expression in RGS5-KO mice indicates activation of the RGS5 promoter, without expression of the RGS5 protein. RGS5-lacking GFP^+^ pericytes were mainly found around blood vessels. GFP was not expressed in any other cell type, in particular not in endothelial cells. Consistent with our previous results, GFP^+^ cells were only found around the capillaries, and their coverage was 2-fold higher in RGS5-KO mice in comparison with their RGS5-HET littermate controls (KO 45.3 ± 5.5%; HET 20.2 ± 10.62%; *P* < 0.0001) (**Fig. 3*A, B***). In addition, we confirmed the increased pericyte coverage of the vasculature in the peri-infarct area using CD13, another pericyte marker (Supplemental Fig. S1). Importantly, we found that only perivascular PDGFR-β^+^ pericytes, not parenchymal PDGFR-β^+^ cells, expressed GFP (Fig. 3*C*). The number of PDGFR-β^+^/GFP^+^ pericytes was 3-fold higher in RGS5-KO mice compared with RGS5-HET mice (KO 394 ± 98 cells/mm^2^; HET 173 ± 65 cells/mm^2^; *P* = 0.001) (Fig. 3*D*), suggesting that lack of RGS5 protein expression leads to higher numbers of pericytes located around the vasculature.

### RGS5 loss retains PDGFR-β signaling at baseline levels after stroke

**Figure 4:**
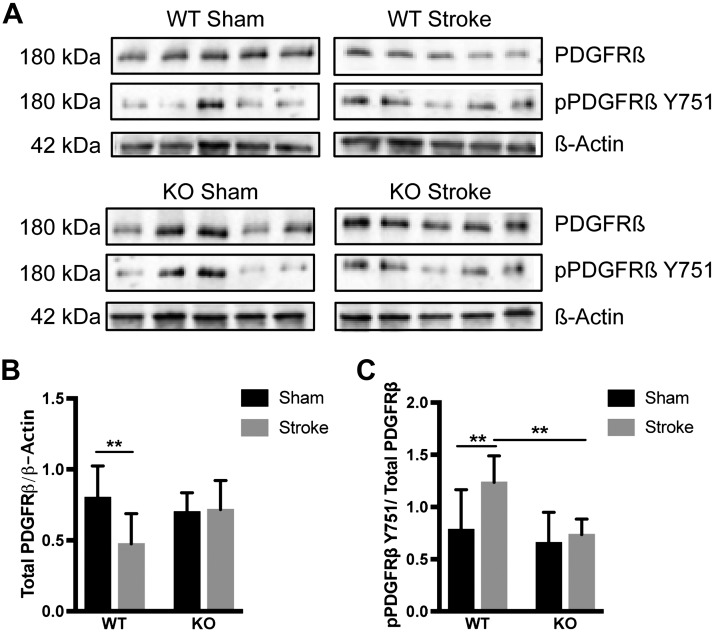
Loss of RGS5 maintains PDGFR-β signaling at baseline level. *A*) Representative Western blot bands of total PDGFR-β (180 kDa), pPDGFR-β Y751 (180 kDa), and β-actin (42 kDa) of WT and KO mice after sham surgery or pMCAO. Figure shows bands from 5 individual mice per group. *B*) Quantification of the PDGFR-β total protein levels normalized to β-actin. *C*) Quantification of the pPDGFR-β Y751 protein levels normalized to the PDGFR-β total protein. *n* = 9 (WT) and 10 (KO); data are represented as means ± sd. **P < 0.01, 2-way ANOVA followed by Tukey’s multiple comparison test.

To assess changes in PDGFR-β signaling activation, we performed Western blot analysis to examine the expression of total PDGFR-β protein and pPDGFR-β (Tyr751) in the infarct area of WT and RGS5-KO mice at 7 d after stroke (**Fig. 4**).

In WT mice, there was a significant decrease in total PDGFR-β protein expression in the ischemic core compared with sham-treated controls. In the ischemic core of RGS5-KO mice, levels of total PDGFR-β protein remained unchanged compared with sham-treated controls (WT_Stroke_ 0.475 ± 0.711, WT_Sham_ 0.802 ± 0.074, *P* = 0.007; KO_Stroke_ 0.717 ± 0.065, KO_Sham_ 0.700 ± 0.045, *P* = 0.998) (Fig. 4*A, B*).

To determine the effect of RGS5 on PDGFR-β phosphorylation, immunoblots for anti–pPDGFR-β (Tyr751) were performed and normalized to the PDGFR-β total protein expression. The ratio of pPDGFR-β (Tyr751) to PDGFR-β total protein was significantly higher in WT mice compared with sham-treated mice (WT_Stroke_ 1.236 ± 0.084; WT_Sham_ 0.784 ± 0.128; *P* = 0.008) but not in RGS5-KO mice (KO_Stroke_ 0.735 ± 0.047; KO_Sham_ 0.658 ± 0.097; *P* = 0.931) (Fig. 4*A, C*), indicating changes in PDGFR-β signaling mediated by RGS5.

### Loss of RGS5 improves blood vessel density in the infarct core

**Figure 5:**
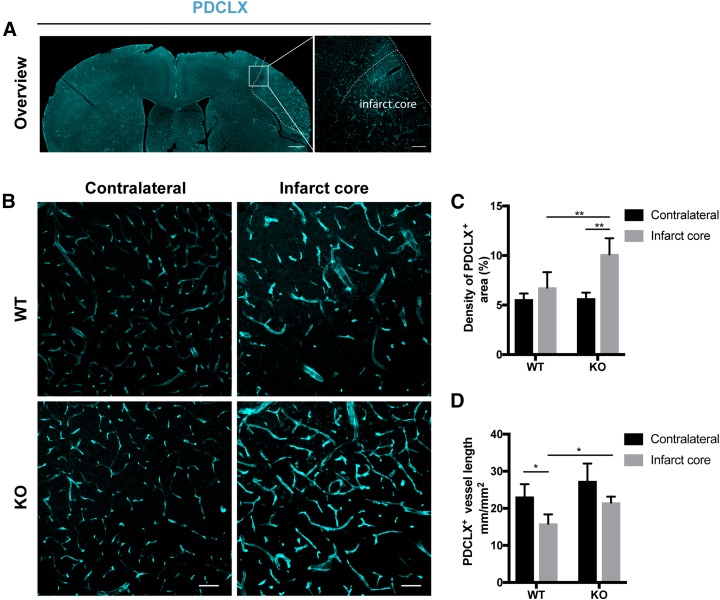
Loss of RGS5 preserves blood vessels in the infarct core. *A*) Overview picture of the vasculature [podocalyxin (PDCLX)] at 7 d after stroke. *B*) Representative confocal images of PDCLX of WT and KO mice 7 d after stroke. Left panel shows the contralateral hemisphere and right panel shows the infarct core. *C*) Quantification of the vascular density of the contralateral hemisphere and the infarct core of WT and RGS5-KO mice. *D*) Quantification of the total vessel length of the contralateral hemisphere and the infarct core of WT and RGS5-KO mice. *n* = 6; data are represented as means ± sd. Scale bars, 500 µm (overview left), 100 µm (overview right), 40 μm (panel B). *P < 0.05, **P < 0.01, 2-way ANOVA followed by Tukey’s multiple comparison test.

We next examined whether RGS5 loss affected the blood vessel morphology in the infarct core (**Fig. 5*A***). In the contralateral hemispheres, there were no morphologic differences in the blood vessels and in vascular density between genotypes (Fig. 5*B*). In the infarct core, however, we found a significant increase in vessel density in RGS5-KO mice as compared with WT mice (KO_contralateral_ 5.7 ± 0.3%, KO_infarct_ 10.1 ± 1.6%, *P* = 0.003; WT_contralateral_ 5.6 ± 0.3%, WT_infarct_ 6.8 ± 1.6%, *P* = 0.622) (Fig. 5*B, C*). The total vessel length was significantly decreased by 35% in the infarct core of WT mice compared with the contralateral hemisphere, whereas the corresponding change in vessel length in RGS5-KO mice was not significant (KO_contralateral_ 27.3 ± 3.3, KO_infarct_ 21.5 ± 1.6 mm/mm^2^, *P* = 0.138; WT_contralateral_ 23.1 ± 2.0 mm/mm^2^, WT_infarct_ 14.9 ± 1.4 mm/mm^2^, *P* = 0.016) (Fig. 5*D*). When we compared differences between the 2 genotypes, the total vessel length was significantly higher in the infarct core of RGS5-KO compared with WT mice (*P* = 0.039) (Fig. 5*D*). Stroke size, however, was not significantly changed between genotypes at 7 d after stroke (Supplemental Fig. S2).

### Loss of RGS5 results in reduced vascular leakage at 7 d after stroke

**Figure 6:**
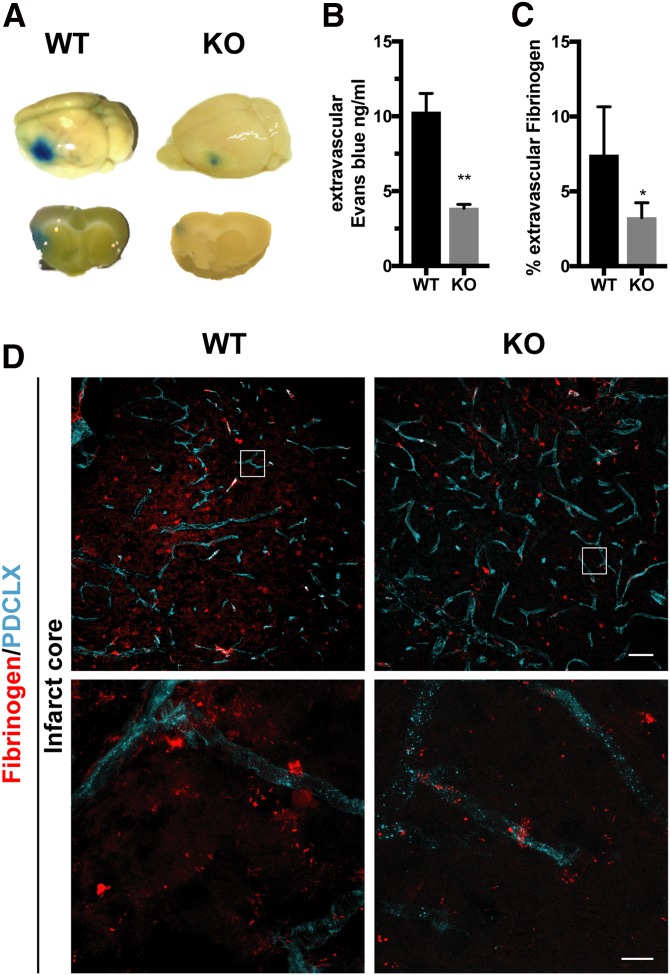
RGS5-KO mice have reduced vascular leakage at 7 d after stroke. *A*) Representative pictures of the whole brain (upper row) and brain sections (lower row) of WT and RGS5-KO mice with Evans blue leakage at 7 d after stroke. *B*) Quantification of Evans blue leakage. *C*) Quantification of extravascular fibrinogen in the infarct core of WT and RGS5-KO mice. *D*) Representative confocal images showing leakage of fibrinogen (red) outside vessels (cyan). Boxes in upper panel indicate where higher magnification in lower panel were taken. PDCLX, podocalyxin, *n* = 5; data are represented as means ± sd. Scale bars, 50 (upper panel) and 10 μm (lower panel). *P < 0.05, **P < 0.01, Student’ *t* test.

Next, we investigated whether the vascular preservation in RGS5-KO mice was accompanied by changes in vascular leakage after stroke. Using intravenously injected Evans blue 7 d after stroke, we observed that Evans blue only leaked into the parenchyma in the ipsilateral hemisphere, not in the contralateral hemisphere (**Fig. 6*A***). There was significantly less vascular leakage in RGS5-KO mice in comparison with WT mice (KO 3.9 ± 0.6 ng/ml; WT 10.3 ± 2.8 ng/ml; *P* = 0.001) (Fig. 6*B*). Staining of fibrinogen confirmed that RGS5-KO mice have reduced vascular leakage in comparison with WT mice (KO 3.2 ± 0.5%; WT 6.7 ± 1.5%; *P* = 0.046).

## DISCUSSION

During the chronic phase of stroke, pericytes are essential to stabilize the remaining and newly formed blood vessels (8–11), but vessel wall–derived pericytes have also been described to migrate into the injured parenchyma and contribute to scar-forming PDGFR-β^+^ cells (4, 5).

Here we demonstrate that lack of RGS5 in brain pericytes has a clear impact on PDGFR-β^+^ pericyte response after stroke. Loss of RGS5 results in increased numbers of perivascular PDGFR-β^+^ cells and a decrease in the density of parenchymal PDGFR-β^+^ cells. This was accompanied by maintenance of PDGFR-β signaling at baseline levels in RGS5-KO mice. We show an increased pericyte coverage that was associated with a preserved vascular density and capillary length as well as reduced vascular leakage.

Using WT and RGS5-KO mice, we provide clear evidence that absence of the protein RGS5 in brain pericytes changes the spatial distribution of pericytes in stroke. In particular, our data demonstrate that loss of RGS5 maintains PDGFR-β^+^ pericytes around the blood vessels. This increased number of perivascular pericytes in the infarct core of RGS5-KO mice may be due to reduced detachment or increased survival of pericytes or both. This is supported by our previous finding that loss of RGS5 results in increased numbers of PDGFR-β^+^ pericytes in the acute phase of stroke (17) and that pericytes express RGS5 before detaching from the blood vessel wall in stroke (14). Loss of RGS5 may interfere with this process and keep pericytes attached to the vessel wall, suggesting that RGS5 protein expression is needed for pericyte detachment. This was reflected in the higher number of perivascular PDGFR-β^+^ pericytes expressing GFP in RGS5-KO mice because GFP expression in these mice indicates activation of the RGS5 promoter without expression of the RGS5 protein.

Previous reports describe that a subpopulation of vessel wall–derived pericytes migrate into the injured parenchyma and become scar-forming PDGFR-β^+^ cells (4, 5, 26). We find a decrease in the density of parenchymal PDGFR-β^+^ cells, suggesting that RGS5 is at least contributing to the fate switch from perivascular pericytes to parenchymal pericytes.

Further, our data show that PDGFR-β signaling is maintained at a baseline level in RGS5-KO mice, whereas there is a significant reduction of PDGFR-β total protein expression in WT mice. Why the PDGFR-β total protein is reduced in WT remains unclear. Immunohistochemistry only provides information on the spatial distribution and density of PDGFR-β^+^ parenchymal cells, whereas the Western blot allows for analysis of protein levels. It is conceivable that the higher number of perivascular pericytes in RGS5-KO mice might be the main contributor to the total PDGFR-β content in the tissue, explaining the differences between RGS5-KO and WT mice. The stabilization in PDGFR-β signaling in RGS5-KO mice may also be implicated in maintaining pericytes around the vessels. PDGFR-β has been shown to be essential for vascular development, and disturbance causes abnormal BBB functions (27–29).

We also determined an increase in the ratio of phosphorylation of PDGFR-β to total PDGFR-β in WT mice, indicating an increased activation of PDGFR-β. Another possible explanation for the reduced protein levels of PDGFR-β in WT mice could be that activation of the PDGFR-β pathway led to internalization of PDGFR-β and thereby reduction in the total PDGFR-β protein. We and others have previously shown that stimulation of PDGFR-β results in decreased surface expression and internalization, which are associated with increased phosphorylation of the receptor (30–34). The increased activation of PDGFR-β as indicated by the increased ratio of pPDGFR-β to total PDGFR-β in WT mice might facilitate pericyte detachment from the vessels and increase the density of parenchymal pericytes. Consistently, phosphorylation at Tyr571 has been characterized to be responsible for PI3K binding (35), which mediates actin reorganization, migration away from the blood vessels, and differentiation (36–38). One hypothesis is that RGS5 may interact with the receptor or the corresponding downstream pathways or both. Indeed, RGS5 has previously been shown to be involved in PDGF-induced ERK phosphorylation (23). In addition, loss of RGS5 has been shown to negatively regulate signaling pathways in other systems [*e.g.*, it has been shown that loss of RGS5 inhibits JNK1/2 and p38 signaling, leading to markedly reversed ischemia-reperfusion–induced cardiomyocyte apoptosis (39)]. However, further studies are needed to dissect the implications of RGS5 on the downstream signaling pathways that are involved in pericyte detachment mechanisms.

In this study, we demonstrate that the switch from a parenchymal pericyte phenotype to a perivascular phenotype has consequences for vascular remodeling in the chronic phase after stroke. The significant increase in perivascular pericytes in RGS5-KO mice not only resulted in higher pericyte coverage but also in improved vascular density and length. It has been shown that pericytes are important modulators of vascular stabilization (8–11). Our findings are supported by previous evidence in a tumor model demonstrating that loss of RGS5 results in vascular normalization due to pericyte maturation (40). Our data add to these findings and demonstrate that loss of RGS5 improves vascular integrity and, associated with this, results in reduced vascular leakage in the chronic phase after stroke. BBB breakdown is a biphasic process (41). We have previously shown reduced BBB leakage in RGS5-KO mice in the acute phase after stroke (17), indicating that loss of RGS5 has an impact on both early and late BBB dysfunction after stroke. The reduced leakage did not result in any changes in stroke size or swelling, which is likely due to the stroke model used in this study, which results in a rather small and focal cortical stroke (25, 42).

The increased vascular density and preserved total vessel length observed in RGS5-KO mice may be due to a protective effect on endothelial cells as a result of the higher number of perivascular remaining pericytes, given that pericytes have been shown to promote endothelial survival (43). In line with this, we have previously shown that loss of RGS5 in the acute phase of stroke results in increased numbers of perivascular pericytes and preserved tight junctions (17). Further, RGS5 overexpression has been demonstrated to inhibit sonic hedgehog–mediated signaling (44), which has been shown to reduce pericyte recruitment and vessel maturation (45), opening the possibility of RGS5 acting on PDGFR-β signaling through sonic hedgehog.

In conclusion, our study suggests that RGS5 is an important modulator of pericyte detachment and involved in the shift from a perivascular phenotype to a parenchymal phenotype. RGS5 may constitute a target to normalize the pericyte response, preserve the vasculature, and reduce vascular leakage in the chronic phase after stroke.

Targeting the pericyte response after stroke is one of many contributing factors to an improved vascular integrity (13, 46). Further studies are needed to address the impact of RGS5 loss on the long-term benefits and other aspects of vascular remodeling.

## Supplementary Material

This article includes supplemental data. Please visit *http://www.fasebj.org* to obtain this information.

## Abbreviations

BBB: blood-brain barrier
GFP: green fluorescent protein
HET: heterozygous
KO: knockout
MCA: middle cerebral artery
NeuN: Neuronal Nuclei
PDGF-BB: platelet-derived growth factor BB
PDGFR-β: platelet-derived growth factor receptor β
pMCAO: permanent MCA occlusion
RGS5: regulator of G-protein signaling 5
WT: wild type
