# Supplementary figures and images for: Regulator of G-protein signaling 5 regulates the shift from perivascular to parenchymal pericytes in the chronic phase after stroke
Source: FASEB J. 2019 Apr 30;33(8):8990–8. doi: 10.1096/fj.201900153R (PMC6662981; doi:10.1096/fj.201900153R)

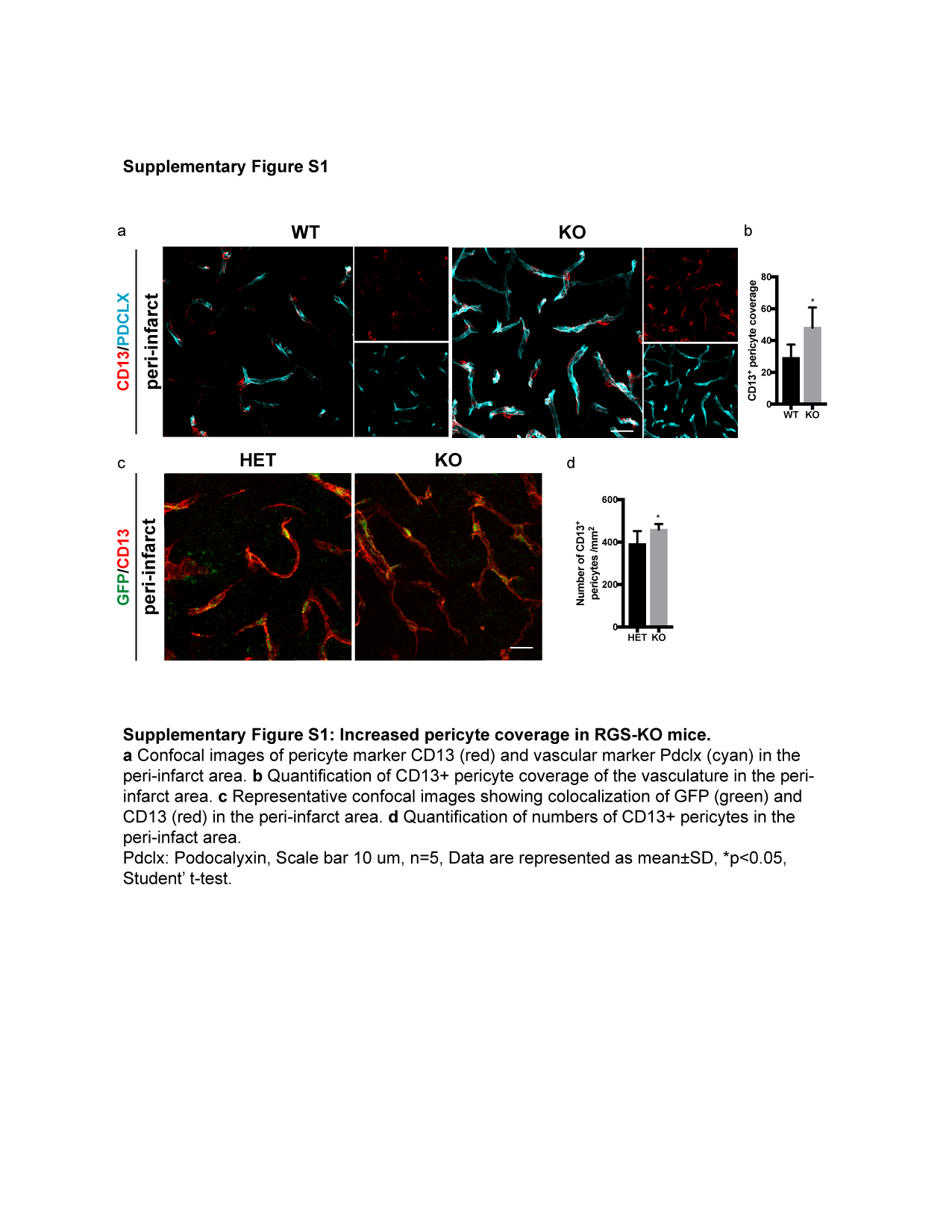

Supplement: Supplementary file 1 [file fj.201900153R.sf1.tif]

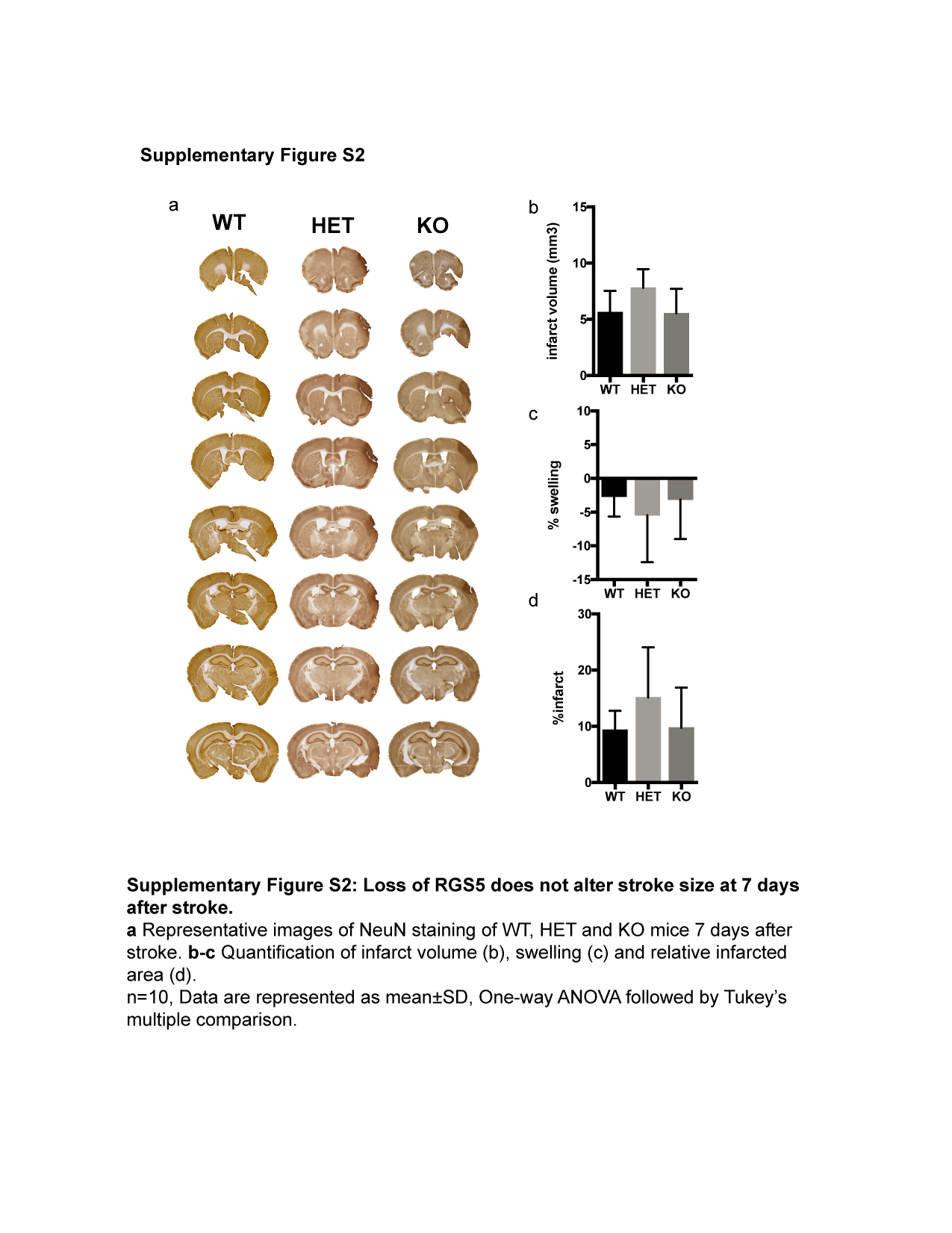

Supplement: Supplementary file 2 [file fj.201900153R.sf2.tif]
